# 10-Eicosanol Alleviates Patulin-Induced Cell Cycle Arrest and Apoptosis by Activating AKT (Protein Kinase B) in Porcine Intestinal Epithelial Cells

**DOI:** 10.3390/ijms25168597

**Published:** 2024-08-07

**Authors:** Chae Hyun Lee, Sangsu Shin, Sang In Lee

**Affiliations:** 1 Department of Animal Science and Biotechnology, Kyungpook National University, Sangju-si 37224, Gyeongsangbuk-do, Republic of Korea; dlcogus9602@naver.com (C.H.L.); sss@knu.ac.kr (S.S.); 2 Research Institute for Innovative Animal Science, Kyungpook National University, Sangju-si 37224, Gyeongsangbuk-do, Republic of Korea

**Keywords:** patulin, intestinal epithelial cell, gene expression profiling, cell cycle arrest, apoptosis, 10-eicosanol

## Abstract

Patulin (PAT) is a fungal toxin prevalent in apples and apple products and associated with several toxic effects, potentially harming multiple organs, including the kidneys, liver, and colon. However, the precise molecular mechanism through which PAT affects the intestines remains comprehensively unclear. Therefore, this study aims to investigate the molecular effects of PAT on the intestinal epithelium. Gene expression profiling was conducted, hypothesizing that PAT induces cell cycle arrest and apoptosis through the PI3K-Akt signaling pathway. Cell cycle analysis, along with Annexin-V and propidium iodide staining, confirmed that PAT induced G2/M phase arrest and apoptosis in IPEC-J2 cells. Additionally, PAT activated the expression of cell cycle-related genes (*CDK1*, *CCNB1*) and apoptosis-related genes (*BCL6*, *CASP9*). Treatment with SC79, an AKT activator, mitigated cell cycle arrest and apoptosis. To identify natural products that could mitigate the harmful effects of PAT in small intestinal epithelial cells in pigs, the high-throughput screening of a natural product library was conducted, revealing 10-Eicosanol as a promising candidate. In conclusion, our study demonstrates that 10-Eicosanol alleviates PAT-induced cell cycle arrest and apoptosis in IPEC-J2 cells by activating AKT.

## 1. Introduction

Mycotoxins—secondary metabolites produced by various fungi—have attracted global attention as significant contaminants owing to their diverse effects on animal and human health [1]. Patulin (PAT), a mycotoxin produced by several molds such as Aspergillus and Penicillium, is most commonly found in apples and apple products; however, PAT can also occur in other foods, including grains, fruits, and vegetables [2,3,4]. PAT exposure can lead to various toxic effects, including genotoxicity, neurotoxicity, immunotoxicity, and carcinogenicity in humans and animals [5,6]. Studies show that PAT exposure causes molecular changes, including oxidative stress, DNA damage, cell cycle arrest, and changes in cellular pathways in organs such as the kidney, liver, colon, immune system, and endocrine glands [7,8]. Despite the growing research on PAT toxicity, its exact molecular mechanism regarding its effect on the intestine remains unclear. Therefore, evaluating the molecular effects of PAT on the intestine is crucial.

The intestinal epithelium lines the inner surface of the digestive tract, interacting with the external environment [9]. The intestinal epithelium acts as a regulated system to control the movement of molecules, supporting diverse intestinal functions [10]. Intestinal epithelial cells (IECs) detect and respond to microbial signals to strengthen barrier integrity and are tightly regulated to ensure appropriate immune responses [11]. Consequently, IECs play crucial immunomodulatory roles, influencing the formation and balance of immune cells in mucosal regions [12]. However, harmful cycles caused by intestinal barrier disruption, uncontrolled cell death, and subsequent inflammation are major contributors to chronic inflammatory and infectious gastrointestinal diseases [13]. Animal studies show that PAT is quickly absorbed from the gastrointestinal tract, causing inflammation and ulceration of the gastric mucosa [14,15]. These findings indicate that PAT negatively affects the gastrointestinal tract. Therefore, comprehensively investigating the cellular response of the intestinal epithelium to PAT is essential.

Various methods, such as physical, chemical, biological, and thermal treatments, are used to reduce mycotoxin levels [16]. However, these methods have disadvantages, including low efficiency, chemical residue issues, and adverse effects on animal health [17,18,19]. Consequently, recent years have witnessed increased interest in utilizing “natural products” to mitigate mycotoxins in a manner that is environmentally and animal-friendly [20,21]. This has led to studies exploring these natural solutions [22,23,24]. Furthermore, 10-Eicosanol, also known as Icosan-10-ol, is a secondary fatty alcohol in which the icosane hydrocarbon chain is substituted with a hydroxyl group at position 10, serving as a plant metabolite [25]. In addition, 10-Eicosanol, a long-chain alcohol, has demonstrated anti-inflammatory properties, reduced platelet aggregation, mitigated endothelial damage, and lowered cholesterol levels in various studies [26,27,28]. Given these properties, 10-Eicosanol is expected to mitigate the detrimental molecular mechanism induced by PAT in intestinal epithelial cells.

Therefore, this study aims to investigate the molecular effects of PAT on intestinal epithelial cells. In this study, gene expression profiling was performed to analyze how PAT affects these cells, focusing on identifying genes significantly upregulated or downregulated by PAT. Building upon this analysis, the study investigates how natural substances affect the intracellular toxicity mechanism of PAT.

## 2. Results

### 2.1. Patulin Reduced Viability in IPEC-J2 Cell

**Figure 1:**
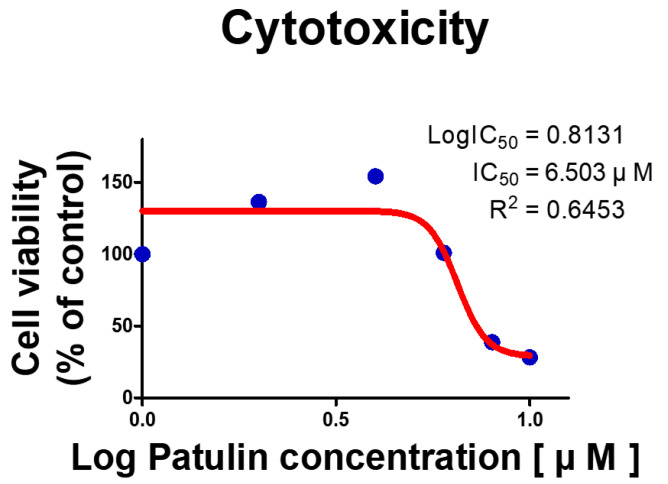
Patulin (PAT) decreased the viability in IPEC-J2 cells, as assessed using the WST-1 cell viability assay. IPEC-J2 cells were exposed to various PAT concentrations (0, 2, 4, 6, 8, and 10 μM) for 24 h, and the half-maximal inhibitory concentration (IC50) was calculated to assess cell viability.

The WST-1 assay was used to evaluate the viability of IPEC-J2 cells treated with PAT at various concentrations (0, 2, 4, 6, 8, and 10 μM) (Figure 1). The half-maximal inhibitory concentration (IC50) was determined to be 6.503 μM. Therefore, a follow-up assessment was performed on IPEC-J2 cells treated with 6.5 μM of PAT for 24 h.

### 2.2. Identification and Validation of Differentially Expressed Genes

**Figure 2:**
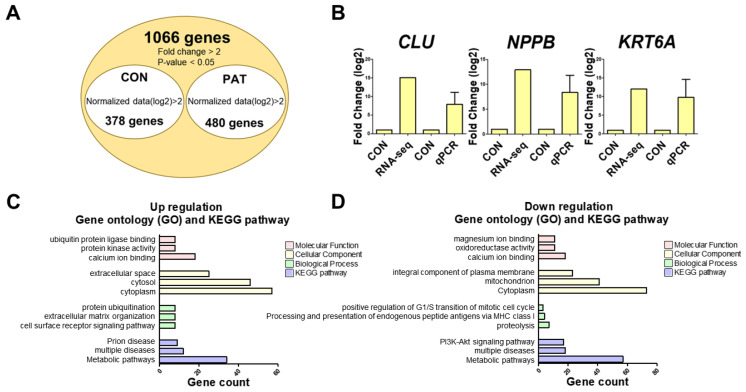
Gene expression profiling of IPEC-J2 cells treated with PAT. (**A**) Venn diagram illustrating genes that are upregulated or downregulated 2-fold compared to those of the control after PAT treatment. The 1066 genes in the Venn diagram suggest a 2-fold change in gene expression, with or without PAT treatment, demonstrating statistical significance (*p* < 0.05). (**B**) The top three upregulated differentially expressed genes (DEGs) (*CLU*, *NPPB*, and *KRT6A*) were verified using real-time quantitative PCR. Error bars represent standard errors from three independent experiments. (**C**) The gene ontology (GO) and KEGG pathway analyses of upregulated DEGs. (**D**) The GO and KEGG pathway analyses of downregulated DEGs.

Follow-up experiments were conducted at a concentration of 6.5 uM, which was the IC50 of PAT determined from cytotoxicity results. The gene expression profile of PAT-treated IPEC-J2 cells was analyzed to identify differentially expressed genes (DEGs). Consequently, 858 DEGs were identified, comprising 480 and 378 upregulated and downregulated genes, respectively (Figure 2A and Tables S1–S8). Additionally, the expression of the top three upregulated genes was confirmed using a real-time quantitative polymerase chain reaction (PCR), and no statistical difference was observed from the RNAseq results (Figure 2B). The 480 upregulated DEGs were categorized through GO analysis into three groups: molecular functions, cellular components, and biological processes (Figure 2C). The “molecular functions” of the upregulated DEGs included “calcium ion binding”, “protein kinase activity”, and “ubiquitin protein ligase binding”. The “cellular components” category encompassed “cytoplasm”, “cytosol”, and “extracellular space”. DEGs classified under “biological processes” were involved in “cell surface receptor signaling pathway”, “extracellular matrix organization”, and “protein ubiquitination”. The Kyoto Encyclopedia of Genes and Genomes (KEGG) analysis identified major upregulated signaling pathways, including “metabolic pathways”, “multiple diseases”, and “prion disease”. The 378 downregulated DEGs were categorized through GO analysis into three groups: molecular functions, cellular components, and biological processes (Figure 2D). Downregulated DEGs within the “molecular function” category included “calcium ion binding”, “oxidoreductase activity”, and “magnesium ion binding”. The “cellular components” category encompassed genes associated with the “cytoplasm”, “mitochondrion”, and “integral component of the plasma membrane”. Additionally, genes classified under “biological process” included “proteolysis”, “antigen processing and presentation of endogenous peptide antigen via MHC class I”, and “positive regulation of the G1/S transition in the mitotic cell cycle”. The KEGG analysis identified the major downregulated signaling pathways as “Metabolic pathways”, “multiple diseases”, and the “PI3K-Akt signaling pathway”.

### 2.3. Patulin Causes Cell Cycle Arrest and Apoptosis

**Figure 3:**
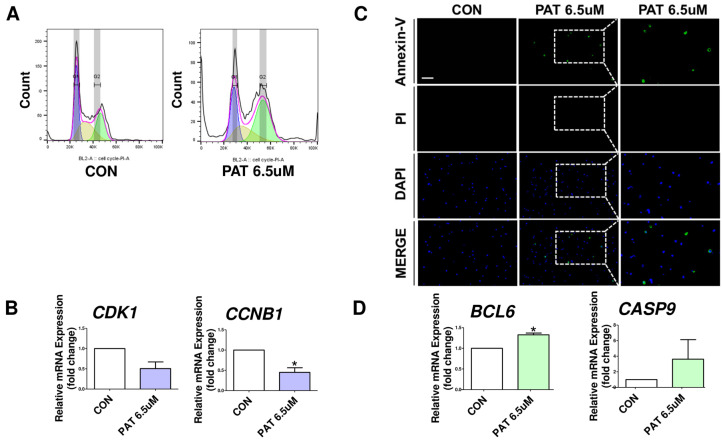
PAT induces cell cycle arrest and apoptosis in IPEC-J2 cells. (**A**) IPEC-J2 cells were treated with PAT (0, 6.5 μM) for 24 h. Cell cycle phases were analyzed using PI staining and flow cytometry. (**B**) The mRNA-expression levels of cell cycle-related genes (*CDK1*, *CCNB1*) after PAT treatment were compared to the negative control (*n* = 3). (**C**) IPEC-J2 cells were treated with PAT (0, 6.5 μM) for 24 h and then stained. Cell staining was performed using single-cell staining (Annexin-V; green, propidium iodide; red). Nuclei were stained with 4’,6-diamidino-2-phenylindole (DAPI; blue). (Scale bar represents 150 μm). (**D**) The mRNA levels of apoptosis-related genes (*BCL6*, *CASP9*) at a PAT concentration of 6.5 μM were compared to the control (*n* = 3). Error bars indicate standard errors (SEs) of triplicate analysis. * *p* < 0.05.

GO analysis confirmed that PAT treatment affects the cell cycle. To further investigate this effect, the cell cycle was quantitatively analyzed using PI staining and flow cytometry. The results show that the percentage of cells in the G2/M phase was higher in PAT-treated cells than in the controls (Figure 3A). Additionally, the mRNA expression levels of cell cycle-related genes (*CDK1*, *CCNB1*) indicate that PAT affected the cell cycle (Figure 3B). To determine whether cell death occurred in PAT exposed to IPEC-J2 cells, we performed cell staining with Annexin-V and propidium iodide (Figure 3C). The results showed that significant apoptosis occurred in IPEC-J2 cells treated with PAT compared to those in the control. The measurement of mRNA expression levels for apoptosis-related genes (*BCL-6* and *CASP9*) revealed significant changes with their expression increasing or decreasing in PAT-treated cells compared to that of the control. (Figure 3D).

### 2.4. Activation of AKT Restores the Cell Cycle and Alleviates Apoptosis

**Figure 4:**
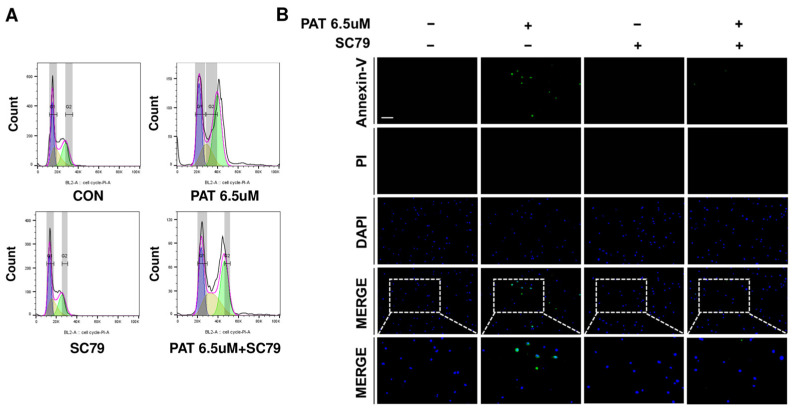
Activation of AKT restores the cell cycle and diminishes apoptosis in IPEC-J2 cells. (**A**) Cell cycle stages and their quantitative measurements were assessed using PI staining and flow cytometry in cells treated with 6.5 μM of PAT and SC79. (**B**) IPEC-J2 cells were treated with 6.5 μM of PAT and SC79 for 24 h. Apoptosis was analyzed using single-cell staining with Annexin-V (green) and propidium iodide (red). Nuclei were stained with 4′,6-diamidino-2-phenylindole (DAPI; blue). (Scale bar represents 150 μm).

KEGG pathway analysis confirmed that the PI3K-Akt signaling pathway was downregulated, suggesting that PAT reduces AKT expression. To test this hypothesis, SC79, as an AKT activator, was used to activate AKT. IPEC-J2 cells were treated with PAT, PAT+SC79, and SC79 alone, and the cell cycle was quantitatively measured using PI staining and flow cytometry. PAT treatment increased the percentage of cells in the G2/M phase compared to those in the control group. However, cells treated with SC79 and PAT showed a lower G2/M phase percentage than those treated with PAT alone (Figure 4A). These findings indicate that SC79 alleviates G2/M phase cell cycle arrest by activating AKT. To further confirm the reduction in apoptosis owing to AKT activity, IPEC-J2 cells were treated with PAT, PAT+SC79, and SC79, then stained with Annexin-V and propidium iodide (Figure 4B). These findings confirmed that apoptosis was significantly reduced when SC79 was added to PAT-treated cells compared to cells treated with PAT alone.

### 2.5. High-Throughput Screening to Discover Natural Products That Mitigate Patulin Toxicity in IPEC-J2 Cells

**Figure 5:**
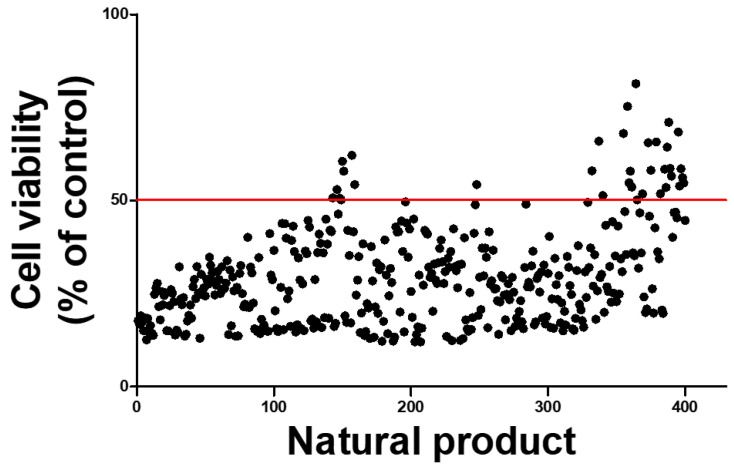
High-throughput screening was conducted to identify potential natural product candidates that could mitigate PAT toxicity in IPEC-J2 cells. Cell viability was assessed in IPEC-J2 cells treated with 6.5 µM of PAT and 20 ng/µL of each natural product for 24 h. The graph shows a red horizontal line representing the IC50 value for cells treated with PAT. The black dots represent the cell viability values for groups treated with PAT and each natural product simultaneously.

To identify potential natural products that might alleviate PAT toxicity, 400 candidates were screened using high-throughput screening (HTS). These products were tested on PAT-treated IPEC-J2 cells to evaluate their influence on cell viability. While most natural products reduced cell viability to <50% owing to PAT toxicity, 34 products were identified that increased cell viability above the IC50 concentration of PAT (Figure 5). Subsequently, the top three natural products with the highest cell viability were selected. Among them, 10-Eicosanol, which most significantly improved cell viability in PAT-treated IPEC-J2 cells, was selected as the most effective natural product for mitigating PAT toxicity.

### 2.6. 10-Eicosanol Mitigates G2/M Phase Arrest and Apoptosis in IPEC-J2 Cells

**Figure 6:**
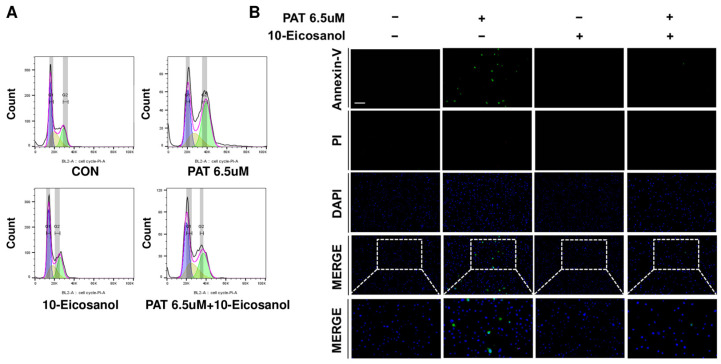
10-Eicosanol restores the cell cycle and reduces apoptosis in IPEC-J2 cells. (**A**) Cell cycle stages and their quantitative measurements were assessed using PI staining and flow cytometry in cells treated with 6.5 μM of PAT and 10-Eicosanol. (**B**) IPEC-J2 cells were treated with 6.5 μM of PAT and 10-Eicosanol for 24 h. Apoptosis was analyzed using single-cell staining with Annexin-V (green) and propidium iodide (red). Nuclei were stained with 4′,6-diamidino-2-phenylindole (DAPI; blue). (Scale bar represents 150 μm).

To determine whether 10-Eicosanol alleviates G2/M phase arrest and apoptosis, cell cycle analysis and apoptosis assessments were performed using Annexin-V and propidium iodide staining and examined under a fluorescence microscope. The treatment of IPEC-J2 cells with PAT significantly increased the percentage of cells in the G2/M phase and decreased those in the G1 phase compared to those in the control. However, co-treatment with PAT and 10-Eicosanol restored the cell cycle distribution to levels similar to that of the control, indicating that 10-Eicosanol mitigated PAT-induced G2/M phase arrest (Figure 6A). Additionally, while PAT treatment induced significant apoptosis in IPEC-J2 cells, the co-administration of 10-Eicosanol markedly decreased PAT-induced apoptosis (Figure 6B). These findings suggest that 10-Eicosanol mitigates PAT-induced G2/M phase arrest and apoptosis in IPEC-J2 cells.

## 3. Discussion

PAT, initially identified as an antibacterial agent named tercinin in 1943, was first discovered in Penicillium griseofulvum. PAT is now recognized as a major food contaminant commonly found in apples and their derivatives, spoiled fruits, and moldy feeds [29]. Previous studies show the potential of PAT to cause cytotoxicity, genotoxicity, and immunotoxicity. PAT exposure induces various cellular responses, including oxidative stress, DNA damage, and the disruption of cellular pathways across multiple organs [5,6,7,8]. Acute toxicity tests on various animals, including rats, chickens, and hamsters, showed that PAT causes significant gastrointestinal damage, such as gastritis, ulcers, necrosis, and hemorrhage in the stomach and jejunum [30]. Additionally, other studies show that PAT increases colonic epithelial permeability, inhibits ion transport, and causes histopathological damage, such as epithelial degeneration [31]. These findings suggest that PAT detrimentally affects the gastrointestinal tract.

In this study, 480 upregulated and 378 downregulated DEGs were identified through gene expression profiling (Figure 2A). The analysis of the GO and KEGG pathways for these DEGs confirmed that PAT is associated with cell cycle regulation, the metabolic pathway, and the PI3K-Akt signaling pathway. The PI3K-AKT pathway is an intracellular signaling pathway that regulates metabolism, proliferation, cell survival, growth, and angiogenesis in response to extracellular signals [32,33,34,35]. Therefore, based on these gene expression-profiling results, PAT was speculated to influence cell cycle regulation and survival through the PI3K-Akt signaling pathway in IPEC-J2.

Cell cycle progression is regulated by CDKs and their regulatory subunits and cyclins, which are activated through the partial phosphorylation of Rb by CDKs and cyclins [36]. Cyclin D/CDK4 functions in the G1 phase, cyclin A/CDK2 in the S phase, and cyclin B/CDC 2 complex in the G2/M phase, all of which are crucial for cell cycle regulation [37]. Previous studies revealed that the oral administration of PAT induces inflammation and damage to the intestinal mucosal, leading to cell cycle arrest upon damage [38,39]. Furthermore, apoptosis is triggered by DNA damage [40,41], representing a conserved mechanism where cells undergo self-destruction [42] to ensure the orderly elimination of defective cells [43]. The abnormal regulation of apoptosis can result in diseases, including autoimmune disorders, neurological conditions, cancer, and stroke [44]. Nonetheless, the molecular mechanisms by which PAT induces cell cycle arrest and apoptosis in IPEC-J2 cells remain unknown and consequently require further investigation. The phosphatidylinositol 3-kinase (PI3K)-protein kinase B (AKT) signaling pathway is crucial for cell survival, proliferation, and apoptosis [45]. PI3K, located downstream of Ras, converts PIP2 to phosphatidylinositol (3,4,5)-phosphate (PIP3), leading to the membrane localization and phosphorylation of Akt [46]. Akt activation promotes cell survival by phosphorylating transcription factors and proteins that regulate cell survival and metabolism, thereby suppressing apoptosis and controlling cell cycle progression [47,48]. Conversely, inhibiting AKT induces mitochondrial dysfunction, leading to various diseases, including apoptosis, cancer, and metabolic syndrome [49,50]. While research on PAT toxicity is increasing, the specific molecular mechanisms by which PAT influences the PI3K-Akt pathway in intestinal epithelial cells, affecting cell cycle regulation and apoptosis, remain unexplored.

IPEC-J2 cells were treated with PAT, and changes in cell cycle stages were quantitatively assessed using PI staining and flow cytometry compared to those of the control group. The results confirm that PAT treatment induces G2/M phase arrest in IPEC-J2 cells. Furthermore, PCR was performed to verify the expression changes in cell cycle-related genes, providing further evidence that PAT induces cell cycle arrest. To confirm PAT-induced cell apoptosis, real-time PCR and Annexin-V analysis were used, revealing that PAT induces apoptosis in IPEC-J2 cells. This finding aligns with previous studies showing that PAT triggers cell apoptosis and cell cycle arrest [41]. Furthermore, treatment with the AKT activator SC79 alleviates PAT-induced apoptosis and cell cycle arrest.

Historically, strategies to mitigate mycotoxin toxicity include using mycotoxin binders, chemical treatments, and irradiation [51,52]. However, conventional physical and chemical detoxification methods for mycotoxins in feed products have several disadvantages, including nutrient loss, chemical residues, and secondary environmental pollution [53,54,55]. Therefore, exploring environmentally friendly and safe methods to inhibit mycotoxin toxicity is essential. Recent studies have shown how natural products can effectively reduce mycotoxin toxicity in an eco-friendly manner [56,57,58]. Therefore, 10-Eicosanol was identified through HTS as a natural product that can mitigate patulin-induced toxicity. 10-Eicosanol is a long-chain alcohol with anti-inflammatory, reduced platelet aggregation, endothelial-protective, and cholesterol-lowering properties [26,27,28]. Applying 10-Eicosanol to IPEC-J2 cells alleviates PAT-induced cell cycle arrest and apoptosis, with effects comparable to those of the AKT activator SC79.

**Figure 7:**
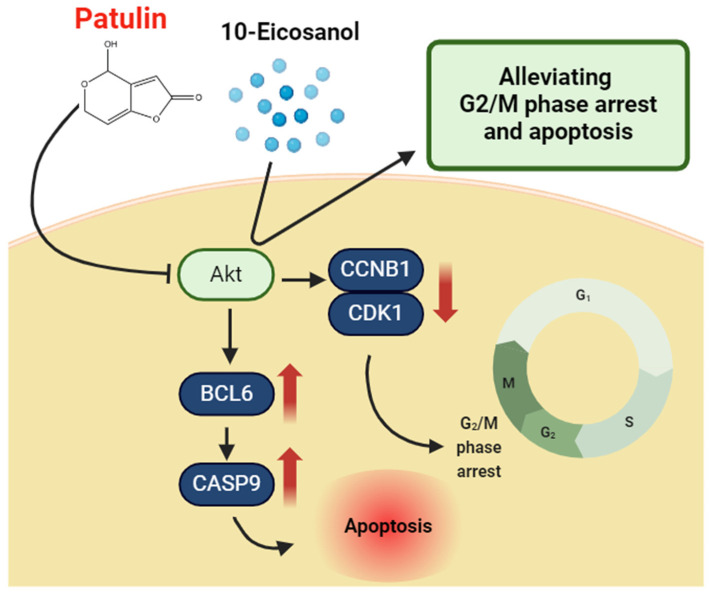
Schematic of the current working hypothesis for 10-Eicosanol to alleviate PAT-mediated cell cycle arrest and apoptosis through AKT activation. PAT induces AKT degradation, which downregulates CCNB1 and CDK1 expression and activates BCL6 and CASP9, leading to cell cycle arrest and apoptosis. Therefore, 10-Eicosanol restores the cell cycle and alleviates apoptosis.

This study, based on gene expression profiling, is the first to show that AKT activation alleviates PAT-induced apoptosis and cell cycle arrest (Figure 7). Additionally, there are no reports on the alleviating effects of 10-Eicosanol on cell cycle and apoptosis. Therefore, this study confirms for the first time that 10-Eicosanol mitigates PAT-induced cell cycle arrest and apoptosis. However, one limitation here is that this study did not directly investigate whether 10-Eicosanol activates AKT directly or acts through an upstream pathway. Therefore, future studies should investigate whether 10-Eicosanol directly interacts with the upstream pathway involved in AKT activation to elucidate its mechanism of action more clearly. Understanding these mechanisms more clearly would offer deeper insights into the cellular signaling processes affected by 10-Eicosanol and could pave the way for developing novel therapeutic strategies.

This study and others clearly show that PAT and other fungal toxins significantly affect cells, even at low concentrations. This highlights the potential harm of PAT as a toxin to animals, including pigs consuming contaminated feed. While allowable limits for PAT are established for apples and apple products, specific standards for PAT in animal feed are unknown. Mycotoxins in feed usually occur in combinations rather than in isolation, which may lead to synergistic effects and complications [59]. Given the elevated risk and significance of PAT as a mycotoxin, further research into its effects is essential. 

Ultimately, these findings may improve our understanding of the molecular mechanisms affected by PAT in the porcine intestinal epithelium. Additionally, this research could offer valuable insights into the harmful effects of PAT-contaminated feed on porcine intestinal epithelial cells. Furthermore, our findings suggest that 10-Eicosanol could be a promising feed additive or plant-based therapeutic for enhancing intestinal health in pigs by reducing PAT-induced cell cycle arrest and apoptosis in porcine intestinal epithelial cells. Consequently, these findings may serve as foundational data for future in vivo studies to more definitively confirm the protective effect of 10-Eicosanol against PAT toxicity.

## 4. Materials and Methods

### 4.1. Cell Culture and Treatment

IPEC-J2 cells (DSMZ, Braunschweig, Germany) are intestinal porcine enterocytes extracted from the jejunum of unsuckled newborn piglets [60]. They were cultured in a 5% CO2 atmosphere at 37 °C using Dulbecco’s Modified Eagle Medium (DMEM) (Thermo Fisher Scientific, Wilmington, DE, USA) supplemented with 10% fetal bovine serum (FBS) and 1% penicillin–streptomycin (PS). PAT (Sigma-Aldrich, St. Louis, MO, USA) was prepared in dimethyl sulfoxide (DMSO) for the treatment of IPEC-J2 cells.

### 4.2. Cell Viability Analysis

Cell viability was assessed 2 h after treatment using WST-1 (Roche Diagnostics GmbH, Mannheim, Germany). The absorbance of the dye was measured with a GloMax Discover Multi-Microplate Reader by subtracting the background wavelength at 600 nm from the reading at 450 nm. IPEC-J2 cells were seeded in a 6-well plate at a density of 1 × $10^{5}\;$ cells per well and incubated for 24 h. Subsequently, the cells were treated with PAT (0, 2, 4, 6, 8, and 10 μM) for an additional 24 h.

### 4.3. Gene-Expression Profiling

IPEC-J2 cells were seeded at a density of 3 × $10^{5}\;$ in 60 pi plates and treated with 6.5 μM PAT for 24 h. RNA extraction was then extracted using the AccuPrep Universal RNA Extraction Kit. RNA quality and quantity were assessed using an Agilent 2100 Bioanalyzer with an RNA 6000 Nano Chip (Agilent Technologies, Amstelveen, The Netherlands) and Thermo Inc ND-2000 Spectrophotometer (Thermo, Wilmington, DE, USA). Libraries were prepared using the QuantSeq 3’ mRNA-Seq Library Prep Kit (Lexogen, Vienna, Austria) following the guidelines of the manufacturer. Briefly, 500 ng of total RNA was hybridized with an oligo-dT primer containing an Illumina-compatible sequence at the 5’ end, followed by reverse transcription. After degrading the RNA template, second-strand synthesis was initiated using random primers with an Illumina-compatible linker sequence at the 5’ end. The double-stranded library was purified using magnetic beads to remove reaction components, which were then amplified to include the full adapter sequence necessary for cluster generation. Finally, the completed library was purified again to eliminate PCR components. High-throughput single-end 75 sequencing was performed using the Next Seq 500 system (Illumina, Inc., San Diego, CA, USA). Gene expression profiling data were analyzed for differential gene expression analysis using Excel V16.0 software. DEGs were analyzed using gene ontology (GO) and KEGG mappers for annotation, integration, and visualization. DEGs with at least a 2-fold change in expression were classified as upregulated or downregulated. 

### 4.4. Cell Cycle Analysis

IPEC-J2 cells were seeded on a 60 mm plate and incubated for 24 h. PAT was then applied for 24 h, after which the cells were washed with phosphate-buffered saline (PBS). The cells were fixed in 80% ethanol on ice, washed with cold PBS, and then stained with propidium iodide (PI). Cell cycle distribution was analyzed using FACSVerse flow cytometry (BD Science, San Jose, CA, USA) and FlowJo V10.9 software. Single cells in G0/G1, S, and G2/M located on the diagonal were analyzed. Furthermore, cells located below this diagonal and remnants with PI fluorescence lower than the subG0 peak were excluded from the analysis.

### 4.5. Annexin-V and Propidium Iodide Staining

After 24 h of PAT treatment on IPEC-J2 cells, which were cultured for 24 h, the cells were harvested and washed with PBS. Following centrifugation and discarding the supernatant, the cells were treated with a 1X Annexin Binding Buffer. Next, 5 μL of Alexa Fluor 488 Annexin-V (Thermo Fisher Scientific, Wilmington, DE, USA) and 1 μL of the 100 mg/mL PI working solution were added. The cells were incubated in the dark for 15 min at room temperature. After incubation, the cells were stained with 3.5 μL of DAPI (Vector Laboratories, Burlingame, CA, USA) and mounted on slides with coverslips. Images were visualized with a fluorescence microscope (Korealabtech, Seongnam-si, Gyeonggi-do, Republic of Korea).

### 4.6. Quantitative Real-Time Polymerase Chain Reaction

IPEC-J2 cells were seeded in 60 mm plates at a density of 3 × $10^{5}$ and incubated for 24 h. The cells were then treated with 6.5 μM of PAT for an additional 24 h. Total RNA was extracted using the AccuPreP Universal RNA Extraction Kit (Cat. No. K-3141, Bioneer, Daejeon, Republic of Korea). Subsequently, cDNA was synthesized from 1 μg of total RNA using the DiaStar™ RT Kit (SolGent, Daejeon, Republic of Korea). Primers for target genes were designed using the Primer3 program (http://frodo.wi.mit.edu) for qPCR analysis. qRT-PCR was conducted under the following conditions: 95 °C for 3 min, followed by 40 cycles of 95 °C for 15 s, 56–58 °C for 15 s, and 72 °C for 15 s. The data were normalized to the expression of glyceraldehyde 3-phosphate dehydrogenase. Fold changes in mRNA expression levels were calculated using the 2^−ΔΔCt^ method. All experiments were conducted in triplicate. Table 1 shows the primer sequences of the genes.

### 4.7. High-Throughput Screening Assay

Four hundred natural products from the National Development Institute for Korean Medicine (Gyeongsan, Republic of Korea) were supplied for HTS analysis. Each product was diluted to a concentration of 1 mg/mL using DMSO. IPEC-J2 cells were cultured in 96-well plates at a density of 5 × $10^{3}\;$ cells per 100 µL for 24 h in media. The cells were then treated with a combination of 6.5 μM of PAT and natural products at a concentration of 20 ng/µL for 24 h. After treatment, cell viability was assessed using WST-1 (Roche Diagnostics GmbH, Mannheim, Germany) for 2 h. The absorbance of the dye was measured using a GloMax Discover Multi-Microplate Reader with the background wavelength of 600 nm subtracted from the measurement at 450.

### 4.8. Statistics

To assess significant differences between the control and treatment groups, data were analyzed using the *t*-test in SAS. Error bars representing the mean ± standard error (SE) were included for triplicate analyses. Statistical significance is indicated with asterisks as follows: * *p* < 0.05 and ** *p* < 0.01, with significance determined using Duncan’s multiple range test.

## Figures and Tables

**Table 1:** Primer utilized for quantitative real-time polymerase chain reaction.

Gene Name	Sequence (5′-3′)	Accession No.
*GAPDH*	Forward: ACACCGAGCATCTCCTGACTReverse: GACGAGGCAGGTCTCCCTAA	NM_001206359
*CLU*	Forward: ATGGATGCCCACCTTCATAGReverse: GCACTTCTCACACTGGTCCTT	NM_213971
*KRT6A*	Forward: CCAGGAGTGGATTTGGTTTTReverse: TCAGAGGGGTGAGGAGATTC	XM_021091688
*NPPB*	Forward: CCGCAGTAGCATCTTCCAAReverse: TGTCAGCCAGGACTTCTCAG	NM_213846
*CDK1*	Forward: TAATAAGCTGGGATCTACCACATCReverse: CGAATGGCAGTACTAGGAACAC	NM_001159304
*CCNB1*	Forward: AGCTAGTGGTGGCTTCAAGGReverse: GCGCCATGACTTCCTCTGTA	NM_001170768
*BCL6*	Forward: GTGTCCTACGGTGCCTTTTTReverse: TGACGCAGAATGTGATGAGA	XM_005657112
*CASP9*	Forward: TACCCTGCCTTACCTTCCACReverse: CTGGTCTTCGGTCATCTGG	XM_013998997

*GAPDH*, glyceraldehyde-3-phosphate dehydrogenase; *CLU*, Clusterin; *KRT6A*, keratin 6A; *NPPB*, natriuretic peptide B; *CDK1*, cyclin dependent kinase 1; *CCNB1*, cyclin B1; *BCL6*, B-cell CLL/lymphoma 6; and *CASP9*, caspase 9.

## Data Availability

Data is contained within the article or Supplementary Material.

## Supplementary Materials

The following supporting information can be downloaded at https://www.mdpi.com/article/10.3390/ijms25168597/s1.
