# Supplementary material for: 10-Eicosanol Alleviates Patulin-Induced Cell Cycle Arrest and Apoptosis by Activating AKT (Protein Kinase B) in Porcine Intestinal Epithelial Cells
Source: Int J Mol Sci. 2024 Aug 7;25(16):8597. doi: 10.3390/ijms25168597 (PMC11354308; doi:10.3390/ijms25168597)
Supplement: Supplementary file 1 [file ijms-25-08597-s001.zip › ijms-3098610-supplementary.pdf]

Table. S1. Biological process in UP-regulation genes (CON vs PAT)

Term	Count	Genes
GO:0007166~cell surface receptor signaling pathway	8	ADGRB3, CREG1, ADGRL1, F2, TSPAN12, CD3E, LOC100522787, GPR157
GO:0030198~extracellular matrix organization	8	ADAMTS2, MMP13, CRISPLD2, COL22A1, TNR, VWA1, ADAMTS9, ADAMTS6
GO:0016567~protein ubiquitination	8	SH3RF3, KLHL25, FBXO2, KLHL7, MAGEF1, RNF182, ABCB11, LOC100623167
GO:0007155~cell adhesion	8	KITLG, ITGAM, ITGAD, LOC100621701, TNR, ITGA5, PCDHA11, FERMT2
GO:0007229~integrin-mediated signaling pathway	6	ADAMTS2, ITGAM, ITGAD, ITGA5, ADAMTS6, FERMT2
GO:0007189~adenylate cyclase-activating G-protein coupled receptor signaling pathway	6	RAMP3, ADORA2A, PTGER3, ADGRL1, LOC100522787, ADCY5
GO:0010506~regulation of autophagy	4	ACER2, DRAM1, MTCL1, TP53INP1
GO:0006631~fatty acid metabolic process	4	GGT5, CPT1A, ABHD5, ABCB11
GO:0010951~negative regulation of endopeptidase activity	4	CSTB, UPTI, TIMP3, LOC396905
GO:0006979~response to oxidative stress	4	NQO1, DUOX1, HMOX1, SELENOK
GO:0030206~chondroitin sulfate biosynthetic process	3	CSGALNACT1, UGDH, XYLT1
GO:0007140~male meiosis	3	SYCP2, ASZ1, TEX14
GO:0034620~cellular response to unfolded protein	3	HSP70.2, HSPB8, HSPA6
GO:0055072~iron ion homeostasis	3	HEPHL1, CFAP69, HMOX1
GO:0071320~cellular response to cAMP	3	AQP9, SLC26A3, HCN1
GO:0055088~lipid homeostasis	3	ABHD4, ABHD5, ASGR2
GO:0031623~receptor internalization	3	GRK3, RAMP3, MX1
GO:0061314~Notch signaling involved in heart development	2	DLL4, SNAI1
GO:1902513~regulation of organelle transport along microtubule	2	HAP1, NEFH
GO:1903589~positive regulation of blood vessel endothelial cell proliferation involved in sprouting angiogenesis	2	FGFBP1, HMOX1
GO:0071243~cellular response to arsenic-containing substance	2	ZFAND2A, HMOX1

Table. S2. Celluar component in UP-regulation genes (CON vs PAT)

Term	Count	Genes
GO:0005737~cytoplasm	57	CDKN1C, SRXN1, ZFAND2A, HSPB8, DUSP19, DOC2B, CLU, NUDT4, NPPB, TRIM9, TUBB6, UCHL1, PNP, CSRP2, HENMT1, ARRDC4, TBCCD1, SGIP1, FST, FBXO2, PTGR1, SDCBP2, TUBA4A, TERB1, BIN2, RILPL2, HECA, SGK1, BRSK1, CSTB, FBLIM1, NDRG1, LDHC, STK31, IRAK2, CREG1, CFAP69, CKB, EPS8L3, DYNC1I1, AAMDC, CAP2, HEXIM1, HSP70.2, MARCKSL1, KLHL25, CAVIN2, MX1, HSPA6, LOC100623167, GADD45G, KITLG, SH3BP5L, GNPDA2, RNF182, RHEBL1, MMACHC
GO:0005829~cytosol	46	USP35, CDA, ITK, CSTB, GRB7, AHCYL2, SRXN1, HSPB8, ABHD5, NDRG1, CLU, ZFYVE28, IL1RL1, UCHL1, PNP, TP53INP2, TP53INP1, CEP170, CKB, CBR3, CNTLN, NQO1, HSP70.2, GABARAPL1, CAVIN2, FBXO2, MX1, HSPA6, PTGR1, PGD, POU2F3, SDCBP2, CIDEC, UGDH, RILPL2, SP4, KLHL7, IRF7, SNAI1, PLIN3, COL21A1, ALDOC, HAP1, SGK1, SQSTM1, FERMT2
GO:0005615~extracellular space	25	CSTB, ITGAM, MTCL1, PROS1, XYLT1, CLU, NPPB, CREG1, SLIT1, TNR, TIMP3, CKB, FST, COL22A1, LOC404703, F2, SYN3, PGC, PROCR, KITLG, CRISPLD2, UPTI, COL21A1, TFF1, UMODL1
GO:0005576~extracellular region	20	FGFBP1, NMB, PROS1, LOC404703, F2, CLU, PGC, KITLG, ADAMTS2, MMP13, CRISPLD2, SPOCK3, SLIT1, TIMP3, VWA1, TFF1, ADAMTS9, LOC396905, ADAMTS6, UMODL1
GO:0005887~integral component of plasma membrane	16	AQP9, LAPTM5, TSPAN12, PCDHA11, ADCY5, SLC6A8, ADORA2A, ADGRB3, SCNN1B, LOC100621701, ADGRL1, LOC100522787, LGR5, SLC26A3, F2RL2, EPHA2
GO:0009986~cell surface	9	DUOX1, ITGAM, RAMP3, TNR, ABCB11, LGR5, CLU, EPHA2, UMODL1
GO:0014069~postsynaptic density	7	PLCB4, MX1, ADGRL1, SYN3, NEFH, DCLK1, CAP2
GO:0031012~extracellular matrix	7	ADAMTS2, MMP13, CRISPLD2, COL22A1, TIMP3, ADAMTS9, ADAMTS6
GO:0030424~axon	7	IL1RL1, KCNB1, MX1, ADGRL1, NEFH, HCN1, SCN1A
GO:0005776~autophagosome	6	GABARAPL1, TP53INP2, TP53INP1, HAP1, SQSTM1, ZFYVE1
GO:0005925~focal adhesion	6	GRB7, IL1RL1, FBLIM1, FERMT2, EPHA2, AIF1L
GO:0005811~lipid particle	5	ABHD4, LPCAT2, PLIN3, ABHD5, ZFYVE1
GO:0008305~integrin complex	3	ITGAM, ITGAD, ITGA5
GO:0097225~sperm midpiece	3	CFAP69, SQSTM1, SLC26A3
GO:0097418~neurofibrillary tangle	2	NEFH, CLU
GO:0043194~axon initial segment	2	NAV1, SCN1A
GO:0042105~alpha-beta T cell receptor complex	2	CREG1, CD3E

Table. S3. Molecular function in UP-regulation genes (CON vs PAT)

Term	Count	Genes
GO:0005509~calcium ion binding	18	PROS1, DOC2B, F2, PCDHA11, AIF1L, DLL4, DUOX1, MMP13, PLCB4, SGCA, CRTAC1, SPOCK3, LOC100621701, LPCAT2, SLIT1, LOC102160959, LOC100522787, UMODL1
GO:0004672~protein kinase activity	8	STK31, BRSK1, GRK3, IRAK2, MMD, TEX14, STK32B, DCLK1
GO:0031625~ubiquitin protein ligase binding	8	HSP70.2, GABARAPL1, UCHL1, HSPA6, LAPTM5, CKB, SQSTM1, CLU
GO:0051787~misfolded protein binding	3	HSP70.2, HSPA6, CLU
GO:0032266~phosphatidylinositol-3-phosphate binding	3	ZFYVE28, PLEKHA4, ZFYVE1
GO:0001882~nucleoside binding	2	CDA, PNP
GO:0042171~lysophosphatidic acid acyltransferase activity	2	ABHD4, ABHD5
GO:0008467~[heparan sulfate]-glucosamine 3-sulfotransferase 1 activity	2	HS3ST4, HS3ST2

Table. S4. KEGG pathway in UP-regulation genes (CON vs PAT)

Term	Count	Genes
ssc01100:Metabolic pathways	34	CDA, AHCYL2, COLGALT2, PDE1C, ATP8, XYLT1, ADCY5, LDHC, PNP, HMOX1, ST8SIA5, GCNT3, ME3, CKB, ST3GAL2, UGT1A6, CBR3, GGT5, CSGALNACT1, NQO1, PCYT1B, DOLK, BPGM, PGD, ACER2, GALC, UGDH, PLCB4, GNPDA2, DHRS9, LPCAT2, ALDOC, ND1, ND2
ssc05022:Pathways of neurodegeneration - multiple diseases	12	GRIA2, TUBB6, UCHL1, PLCB4, ATP8, ND1, HAP1, SQSTM1, NEFH, ZFYVE1, ND2, TUBA4A
ssc05020:Prion disease	9	HSP70.2, TUBB6, CREB3L3, ATP8, HSPA6, ND1, HSPA2, ND2, TUBA4A
ssc05016:Huntington disease	9	GRIA2, TUBB6, PLCB4, CREB3L3, ATP8, ND1, HAP1, ND2, TUBA4A
ssc04610:Complement and coagulation cascades	8	PROCR, ITGAM, PROS1, C3AR1, F2, CLU, F2RL2, LOC100515090
ssc05012:Parkinson disease	8	TUBB6, UCHL1, ADORA2A, ATP8, ND1, ND2, TUBA4A, ADCY5
ssc04915:Estrogen signaling pathway	7	HSP70.2, PLCB4, CREB3L3, HSPA6, TFF1, HSPA2, ADCY5
ssc05162:Measles	7	HSP70.2, HSPA6, MX1, IRF7, HSPA2, CD3E, LOC100515090
ssc05417:Lipid and atherosclerosis	7	HSP70.2, PLCB4, HSPA6, IRF7, LY96, HSPA2, POU2F3
ssc04723:Retrograde endocannabinoid signaling	6	GRIA2, PLCB4, ND1, ABHD6, ND2, ADCY5
ssc04141:Protein processing in endoplasmic reticulum	6	HSP70.2, FBXO2, UBE2D3, LOC100520183, HSPA6, HSPA2
ssc05134:Legionellosis	5	HSP70.2, LOC100514912, ITGAM, HSPA6, HSPA2
ssc04918:Thyroid hormone synthesis	5	DUOX1, PLCB4, CREB3L3, ASGR2, ADCY5
ssc04978:Mineral absorption	4	HEPHL1, HMOX1, SLC26A3, FTL
ssc04923:Regulation of lipolysis in adipocytes	4	PTGER3, ABHD5, CIDEC, ADCY5
ssc04213:Longevity regulating pathway - multiple species	4	HSP70.2, HSPA6, HSPA2, ADCY5
ssc00860:Porphyrin metabolism	3	HEPHL1, HMOX1, UGT1A6

Table. S5. Biological process in DOWN-regulation genes (CON vs PAT)

Term	Count	Genes
GO:0006508~proteolysis	7	MIPEP, ERAP2, CPQ, ERAP1, CTSH, CAPN1, APEH
GO:0019885~antigen processing and presentation of endogenous peptide antigen via MHC class I	4	ERAP2, ERAP1, TAP2, TAPBP
GO:0071294~cellular response to zinc ion	4	MT1A, MT-2B, HVCN1, MT1D
GO:0043171~peptide catabolic process	4	ERAP2, CPQ, ERAP1, LTA4H
GO:0006120~mitochondrial electron transport, NADH to ubiquinone	4	NDUFS8, NDUFA10, COQ9, NDUFV1
GO:0002376~immune system process	4	IRF1, PSMB10, PSMB8, PSMB9
GO:0051603~proteolysis involved in cellular protein catabolic process	4	CTSH, PSMB10, PSMB8, PSMB9
GO:0006006~glucose metabolic process	4	H6PD, IGF2, PGM5, HK2
GO:0006633~fatty acid biosynthetic process	4	SCD, FASN, LIPG, HSD17B8
GO:0006635~fatty acid beta-oxidation	4	ACOXL, ECHS1, E4F1, HSD17B10
GO:0032981~mitochondrial respiratory chain complex I assembly	4	NDUFS8, NDUFS7, NDUFA10, NDUFAF3
GO:0008652~cellular amino acid biosynthetic process	3	PYCR1, BCAT1, BCAT2
GO:0006627~protein processing involved in protein targeting to mitochondrion	3	MIPEP, IMMP2L, PMPCA
GO:0010273~detoxification of copper ion	3	MT1A, MT-2B, MT1D
GO:0045926~negative regulation of growth	3	MT1A, MT-2B, MT1D
GO:0071280~cellular response to copper ion	3	MT1A, MT-2B, MT1D
GO:0006414~translational elongation	3	EEF1B2, EEF2, TUFM
GO:0001678~cellular glucose homeostasis	3	PIK3R2, NUCKS1, HK2
GO:0045737~positive regulation of cyclin-dependent protein serine/threonine kinase activity	3	CCND2, CCND1, PKD2
GO:0045429~positive regulation of nitric oxide biosynthetic process	3	DDAH1, DDAH2, PKD2
GO:0034332~adherens junction organization	3	CDH6, EPHA4, CDH24
GO:0071276~cellular response to cadmium ion	3	MT1A, MT-2B, MT1D
GO:0070371~ERK1 and ERK2 cascade	3	GRB10, CTSH, SOX9
GO:1900087~positive regulation of G1/S transition of mitotic cell cycle	3	CCND2, CCND1, ADAMTS1
GO:0006882~cellular zinc ion homeostasis	3	MT1A, MT-2B, MT1D
GO:0070059~intrinsic apoptotic signaling pathway in response to endoplasmic reticulum stress	3	TRIB3, CHAC1, ATF4

GO:0006772~thiamine metabolic process	2	THTPA, TPK1
GO:0009082~branched-chain amino acid biosynthetic process	2	BCAT1, BCAT2
GO:0009081~branched-chain amino acid metabolic process	2	BCAT1, BCAT2
GO:0035502~metanephric part of ureteric bud development	2	FOXJ1, PKD2
GO:0070221~sulfide oxidation, using sulfide:quinone oxidoreductase	2	TSTD1, TST
GO:0098708~glucose import across plasma membrane	2	SLC5A1, SLC5A2
GO:0035377~transepithelial water transport	2	SLC5A1, CFTR
GO:0045607~regulation of auditory receptor cell differentiation	2	MYCL, MYCN
GO:0009063~cellular amino acid catabolic process	2	ENOSF1, ETFB
GO:0000052~citrulline metabolic process	2	DDAH1, DDAH2
GO:0006550~isoleucine catabolic process	2	HSD17B10, BCAT2
GO:0051702~interaction with symbiont	2	GPX1, FN1
GO:0006574~valine catabolic process	2	ALDH6A1, HIBADH

Table. S6. Celluar component in DOWN-regulation genes (CON vs PAT)

Term	Count	Genes
GO:0005737~cytoplasm	73	RNH1, NDUFA10, NUCKS1, APEH, DNPH1, IPO4, NUBP2, UXT, RBM3, THTPA, CCND2, CCND1, KYNU, STK16, GRB10, ROPN1L, CHAC1, CAPN1, GYG2, AASS, GARNL3, EPHA4, TRPC6, TRAPPC2L, TSTD1, PABPC4, CIRBP, PASK, WDR35, DUSP6, TUBB2B, MT1A, IRF1, PSME1, ELMO3, NSMF, MT1D, ATF4, CAMK2D, CDCA7, HDAC9, PSMB10, ABLIM1, ABLIM3, MT-2B, CBS, PCBP4, APBB2, LTA4H, CFAP65, MBNL3, DTD1, S100A11, ISOC2, TRAPPC2, EGR1, ERAP2, BDNF, ERAP1, KCNIP4, PSMB8, MAPK12, BSPRY, PSMB9, PFKL, PSAT1, FABP6, IMPDH2, WNK3, TCF3, SSBP2, PPIL6, BEX5
GO:0005739~mitochondrion	41	MIPEP, PIF1, ECHS1, HIBADH, MTFP1, MRPS34, HK2, ACOXL, ADAM28, ABHD11, MTG1, NTHL1, KYNU, PMPCA, D2HGDH, ROMO1, E4F1, CAPN1, PUSL1, VARS2, AASS, TGM2, PCK2, STPG1, ACAD8, GSTK1, GPX1, IDH3G, BDNF, IDH2, PYCR1, ACSL5, COQ9, WARS2, TUFM, ALDH6A1, TFAP4, TST, DDAH2, BCAT1, BCAT2
GO:0005887~integral component of plasma membrane	23	GABRA2, EPHA4, GABRP, TRPC6, SLC24A3, SEMA3C, TM2D1, SEMA3D, HVCN1, ABCA4, HTR1D, PKD2, NCMAP, GHR, UPK1B, SLC6A9, ANKH, SLC16A7, ADAM8, ASIC3, FGFR3, CFTR, SLC19A1
GO:0005783~endoplasmic reticulum	22	PHTF2, FIS1, ERAP2, CPQ, LOC100517372, RPL34, INSIG1, TRAPPC5, ABCA4, TMEM132A, ACSL5, ZDHHC24, EEF1B2, RCN1, TRAPPC6A, CREB3L1, SERPINH1, AGR2, APBB2, FAM20A, FGFR3, TGM2
GO:0005743~mitochondrial inner membrane	9	NDUFA7, MTG1, NDUFAF3, PMPCA, ROMO1, COQ9, SLC25A5, SLC25A22, SLC25A6
GO:0000785~chromatin	9	UXT, EGR1, CCND2, IRF1, TRPS1, CREB3L1, NUCKS1, IPO4, TGM2
GO:0005604~basement membrane	7	LAMA5, ADAMTS1, LAMA1, ATRNL1, FN1, COL4A5, AGRN
GO:0005769~early endosome	7	EPHA4, SNX17, LIPG, APBB2, SLC5A1, VPS28, CFTR
GO:0005930~axoneme	5	SPAG6, DNAJB13, DRC1, WDR35, CFAP45
GO:0030008~TRAPP complex	4	TRAPPC2, TRAPPC6A, TRAPPC2L, TRAPPC5
GO:0005747~mitochondrial respiratory chain complex I	4	NDUFS8, NDUFS7, NDUFA10, NDUFV1
GO:0001725~stress fiber	4	ABLIM1, ABLIM3, LIMCH1, PGM5
GO:0036464~cytoplasmic ribonucleoprotein granule	4	GHR, TSTD1, CTSH, CKAP4
GO:0005839~proteasome core complex	3	PSMB10, PSMB8, PSMB9
GO:0032127~dense core granule membrane	2	VPS13C, ADAM8
GO:1990111~spermatoproteasome complex	2	PSMB10, PSMB8

Table. S7. Molecular function in DOWN-regulation genes (CON vs PAT)

Term	Count	Genes
GO:0005509~calcium ion binding	18	RYR2, KCNIP4, VLDLR, PKD2, THBS3, DLK2, CDH6, MYL5, RCN1, EFEMP1, SYT10, CDH24, ADAM8, CAPN1, S100A14, AGRN, S100A11, TGM2
GO:0016491~oxidoreductase activity	11	GSTK1, HIBADH, SCD, AIFM2, LOC100517372, FASN, D2HGDH, HSD17B10, HSD17B8, PAOX, BCAT2
GO:0000287~magnesium ion binding	11	THTPA, PIF1, IDH3G, ENOSF1, IDH2, PGM5, ATP10A, MAP2K7, MAPK12, PLA1A, NME1
GO:0004222~metalloendopeptidase activity	6	MIPEP, ADAM28, YBEY, ADAMTS1, ADAMTSL3, ADAM8
GO:0051287~NAD binding	5	HIBADH, CTBP1, IDH3G, IDH2, NDUFV1
GO:0051539~4 iron, 4 sulfur cluster binding	5	NDUFS8, NDUFS7, NTHL1, NDUFV1, NUBP2
GO:0004175~endopeptidase activity	5	ADAM28, CTSH, PSMB10, PSMB8, PSMB9
GO:0016853~isomerase activity	4	ECI1, DSEL, FUOM, CFTR
GO:0008137~NADH dehydrogenase (ubiquinone) activity	4	NDUFS8, NDUFS7, NDUFA10, NDUFV1
GO:0008237~metallopeptidase activity	4	ADAM28, AMZ2, ADAM8, LTA4H
GO:0005254~chloride channel activity	4	GABRA2, GABRP, SLC1A4, CFTR
GO:0019911~structural constituent of myelin sheath	3	MAL2, MBP, NCMAP
GO:0004298~threonine-type endopeptidase activity	3	PSMB10, PSMB8, PSMB9
GO:0005520~insulin-like growth factor binding	3	IGFBP5, KAZALD1, IGF1R
GO:0070006~metalloaminopeptidase activity	3	ERAP2, ERAP1, LTA4H
GO:0045499~chemorepellent activity	3	SEMA3C, SEMA3D, FLRT3
GO:0003746~translation elongation factor activity	3	EEF1B2, EEF2, TUFM
GO:0052656~L-isoleucine transaminase activity	2	BCAT1, BCAT2
GO:0050337~thiosulfate-thiol sulfurtransferase activity	2	TSTD1, TST
GO:0052654~L-leucine transaminase activity	2	BCAT1, BCAT2
GO:0050048~L-leucine:2-oxoglutarate aminotransferase activity	2	BCAT1, BCAT2
GO:0052655~L-valine transaminase activity	2	BCAT1, BCAT2
GO:0015151~alpha-glucoside transmembrane transporter activity	2	SLC5A1, SLC5A2
GO:0016403~dimethylargininase activity	2	DDAH1, DDAH2
GO:0004084~branched-chain-amino-acid transaminase activity	2	BCAT1, BCAT2
GO:0048763~calcium-induced calcium release activity	2	RYR2, PKD2
GO:0005471~ATP:ADP antiporter activity	2	SLC25A5, SLC25A6
GO:0004735~pyrroline-5-carboxylate reductase activity	2	PYCR1, PYCR3
GO:0050833~pyruvate transmembrane transporter activity	2	MPC1L, SLC16A7
GO:0044594~17-beta-hydroxysteroid dehydrogenase (NAD+) activity	2	HSD17B10, HSD17B8
GO:0005315~inorganic phosphate transmembrane transporter activity	2	SLC25A3, ANKH
GO:0047035~testosterone dehydrogenase (NAD+) activity	2	HSD17B10, HSD17B8
GO:0055056~D-glucose transmembrane transporter activity	2	SLC5A1, SLC5A2

Table. S8. KEGG pathway in DOWN-regulation genes (CON vs PAT)

Term	Count	Genes
ssc01100:Metabolic pathways	57	B4GALT2, HIBADH, NDUFA10, PFAS, SAT1, HK2, THTPA, KYNU, ENOSF1, LIPG, CHAC1, GYG2, AASS, ACAD8, CHST9, GSTK1, GPX1, ACSL5, ALG12, POMGNT1, NME1, NDUFS8, NDUFS7, TST, ITPKA, BCAT1, BCAT2, PFKFB2, ECHS1, H6PD, HSD17B10, HSD17B8, CBS, ALDH3B1, HYI, LTA4H, ATP6V1G3, NDUFV1, PCK2, NDUFA7, TPK1, IDH3G, IDH2, C1GALT1, MBOAT2, PYCR1, ASNS, B3GALT5, PYCR3, UCKL1, ALDH6A1, GALE, PFKL, PSAT1, SCD, IMPDH2, FASN
ssc05022:Pathways of neurodegeneration - multiple diseases	18	RYR2, CAMK2D, NDUFA7, GPX1, BDNF, NDUFA10, HSD17B10, MAPK12, IL1A, TUBB2B, NDUFS8, NDUFS7, CAPN1, SLC25A5, MAP2K7, NDUFV1, SLC25A6, ATF4
ssc04151:PI3K-Akt signaling pathway	17	LAMA5, LAMA1, BDNF, VEGFB, FN1, IGF2, PIK3R2, IGF1R, THBS3, GHR, CCND2, CCND1, CREB3L1, COL4A5, FGFR3, ATF4, PCK2
ssc05020:Prion disease	15	EGR1, RYR2, NDUFA7, NDUFA10, PIK3R2, MAPK12, IL1A, TUBB2B, NDUFS8, NDUFS7, CREB3L1, SLC25A5, NDUFV1, SLC25A6, ATF4
ssc05016:Huntington disease	15	NDUFA7, GPX1, BDNF, NDUFA10, TUBB2B, NDUFS8, NDUFS7, CREB3L1, POLR2G, SLC25A5, MAP2K7, NDUFV1, POLR2L, SLC25A6, TGM2
ssc05010:Alzheimer disease	14	NDUFA7, NDUFA10, PIK3R2, HSD17B10, IL1A, TUBB2B, NDUFS8, NDUFS7, CAPN1, SLC25A5, MAP2K7, NDUFV1, SLC25A6, ATF4
ssc05415:Diabetic cardiomyopathy	12	RYR2, CAMK2D, NDUFA7, NDUFS8, NDUFS7, NDUFA10, MPC1L, PIK3R2, SLC25A5, NDUFV1, MAPK12, SLC25A6
ssc04152:AMPK signaling pathway	11	PFKFB2, PFKL, CCND1, SCD, CREB3L1, FASN, PIK3R2, EEF2, CFTR, IGF1R, PCK2
ssc04510:Focal adhesion	11	LAMA5, MYL5, CCND2, CCND1, LAMA1, VEGFB, FN1, COL4A5, PIK3R2, IGF1R, THBS3
ssc01230:Biosynthesis of amino acids	10	PFKL, CBS, IDH3G, PSAT1, IDH2, PYCR1, ASNS, PYCR3, BCAT1, BCAT2
ssc05166:Human T-cell leukemia virus 1 infection	10	IL15RA, EGR1, CCND2, CCND1, CREB3L1, PIK3R2, TCF3, SLC25A5, SLC25A6, ATF4
ssc05208:Chemical carcinogenesis - reactive oxygen species	10	NDUFA7, NDUFS8, NDUFS7, NDUFA10, PIK3R2, SLC25A5, MAP2K7, NDUFV1, MAPK12, SLC25A6
ssc05012:Parkinson disease	10	TUBB2B, CAMK2D, NDUFA7, NDUFS8, NDUFS7, NDUFA10, SLC25A5, NDUFV1, SLC25A6, ATF4
ssc04218:Cellular senescence	9	IL1A, CCND2, CCND1, PIK3R2, CAPN1, SLC25A5, E2F5, MAPK12, SLC25A6
ssc04932:Non-alcoholic fatty liver disease	9	IL1A, NDUFA7, NDUFS8, NDUFS7, NDUFA10, PIK3R2, NDUFV1, MAPK12, ATF4
ssc04360:Axon guidance	9	EPHA4, MYL5, CAMK2D, ABLIM1, TRPC6, SEMA3C, ABLIM3, SEMA3D, PIK3R2
ssc04714:Thermogenesis	9	NDUFA7, NDUFS8, NDUFS7, CREB3L1, NDUFA10, ACSL5, NDUFAF3, NDUFV1, MAPK12
ssc04933:AGE-RAGE signaling pathway in diabetic complications	8	IL1A, EGR1, CCND1, VEGFB, FN1, COL4A5, PIK3R2, MAPK12
ssc01200:Carbon metabolism	8	PFKL, ALDH6A1, H6PD, ECHS1, IDH3G, PSAT1, IDH2, HK2
ssc04926:Relaxin signaling pathway	8	GNA15, CREB3L1, VEGFB, COL4A5, PIK3R2, MAP2K7, MAPK12, ATF4
ssc04723:Retrograde endocannabinoid signaling	8	GABRA2, GABRP, NDUFA7, NDUFS8, NDUFS7, NDUFA10, NDUFV1, MAPK12

ssc00280:Valine, leucine and isoleucine degradation	7	ACAD8, ALDH6A1, ECHS1, HIBADH, BCAT1, HSD17B10, BCAT2
ssc04512:ECM-receptor interaction	6	LAMA5, LAMA1, FN1, COL4A5, AGRN, THBS3
ssc05222:Small cell lung cancer	6	LAMA5, CCND1, LAMA1, FN1, COL4A5, PIK3R2
ssc05146:Amoebiasis	6	LAMA5, GNA15, LAMA1, FN1, COL4A5, PIK3R2
ssc04668:TNF signaling pathway	6	IRF1, CREB3L1, PIK3R2, MAP2K7, MAPK12, ATF4
ssc04722:Neurotrophin signaling pathway	6	CAMK2D, BDNF, PIK3R2, MAP2K7, MAPK12, ATF4
ssc02010:ABC transporters	5	ABCB1, ABCA4, TAP2, ABCB8, CFTR
ssc00270:Cysteine and methionine metabolism	5	TST, CBS, PSAT1, BCAT1, BCAT2
ssc01212:Fatty acid metabolism	5	ECHS1, SCD, FASN, ACSL5, HSD17B8
ssc05230:Central carbon metabolism in cancer	5	PFKL, IDH2, PIK3R2, FGFR3, HK2
ssc04917:Prolactin signaling pathway	5	CCND2, CCND1, IRF1, PIK3R2, MAPK12
ssc04912:GnRH signaling pathway	5	EGR1, CAMK2D, MAP2K7, MAPK12, ATF4
ssc01210:2-Oxocarboxylic acid metabolism	4	IDH3G, IDH2, BCAT1, BCAT2
ssc00052:Galactose metabolism	4	B4GALT2, PFKL, GALE, HK2
ssc00051:Fructose and mannose metabolism	4	PFKFB2, PFKL, ENOSF1, HK2
ssc03050:Proteasome	4	PSME1, PSMB10, PSMB8, PSMB9
ssc04978:Mineral absorption	4	MT1A, MT-2B, SLC5A1, LOC100739663
ssc00533:Glycosaminoglycan biosynthesis - keratan sulfate	3	B4GALT2, B3GNT7, CHST1
ssc00061:Fatty acid biosynthesis	3	FASN, ACSL5, HSD17B8
ssc00290:Valine, leucine and isoleucine biosynthesis	2	BCAT1, BCAT2
